# Calculating and analyzing time delay in zigzag graphene nanoscrolls based complementary metal-oxide-semiconductors

**DOI:** 10.1038/s41598-024-58593-8

**Published:** 2024-04-19

**Authors:** Ali Sadeqian, Mohammad Taghi Ahmadi, Morteza Bodaghzadeh, Amir Musa Abazari

**Affiliations:** 1 Nanotechnology Research Center, Nanoelectronics Group, Physics Department, Urmia University, Urmia, 57147 Iran; 2 Department of Mechanical Engineering, Faculty of Engineering, Urmia University, Urmia, Iran

**Keywords:** Graphene nanoscroll, Carrier mobility, FET, Current–voltage characteristic, Time delay, Electronic properties and materials, Structural properties, Mechanical and structural properties and devices

## Abstract

Graphene Nano Scrolls (GNSs) and Zigzag graphene nanoscrolls (ZGNSs) are semi-one-dimensional materials with exceptional electrical and optical properties, making them attractive to be used in nanoelectronics and complementary metal–oxide–semiconductor (CMOS) technology. With in CMOS device technology, time delay is a crucial issue in the design and implementation of CMOS based ZGNSs. Current paper focus is on ZGNSs application in the channel area of metal–oxide–semiconductor field-effect transistors (MOSFETs) in CMOS technology. We studied analytically, the importance of different parameters on time delay reduction, resulting in faster switching and higher frequency in integrated circuits (ICs). The results of this research demonstrates that, the ZGNS-based CMOS proves considerable variations in the current due to the geometrical parameters, such as chirality number, channel length, and nanoscroll length which can be engineered to produce faster ICs.

## Introduction

**Figure 1:**
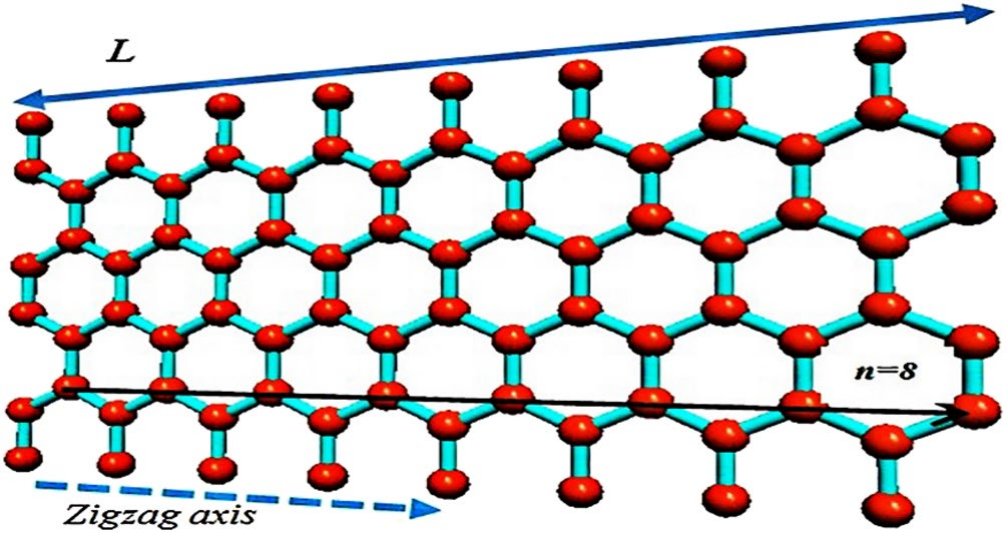
A graphene sheet when scrolled along the zigzag axis, nanoscroll can be obtained; in this figure n = 8.

The exceptional and unique physical properties like electrical, mechanical, magnetic, optical and etc. in sensing or actuating methods, play an important role in the science and industry^1–5^ which motivates the researchers to investigate new materials for future technology. Graphene nanoscroll (GNS) has exceptional electrical and optical properties, making it attractive for researchers in nanoelectronics applications^6–11^. Comparing GNSs with carbon nanotubes (CNTs), Boron Nitro silicon (Si 2 BN)^12^ or Penta grapheme^13^ shows that the larger diameter of GNSs and more appropriate accessibility into the interlayer positions of grapheme nanosheets are provided for foreign molecules with unsealed edges/top ends which simplifies the electrolyte infiltration and axial electron transfer^10,11,14^. As shown in Fig. 1, GNS can be made by rolling a graphene sheet to form an open cylindrical structure. GNS with a tubular structure similar to that of the open CNT has various structures like armchair (n, n), zigzag (n, 0), and chiral (n, m)^6,15^. As the borders of scrolled sheets of GNSs are not connected, they contain an open morphology dissimilar to multi-walled nanotubes^16^. Two dissident energy contributions are achieved by controlling scroll configuration and increasing the elastic energy by bending the graphene sheets to decrease free energy and interactions in the overlapping areas of the graphene sheet^17^. Unrolling back of GNSs into their planar morphology is prevented by the π–π interactions and van der Waals between the overlapping layers^9^.

These exceptional structural specifications result in unique mechanical, electronic, thermal, and anticorrosive properties of GNS and make them appealing candidates for applications in batteries, ion channels, sensors, supercapacitors, and electronic devices^18,19^. Quick and efficient carbon nanoscroll based device fabrication with gate/temperature dependent transport mechanism has been reported^20^, however carbon nano scroll based Complementary metal–oxide–semiconductor (CMOS)fabrication still remain as a challenging research and need to be explored.

Complementary metal–oxide–semiconductor, which is the part of integrated circuits (ICs), and one of the most important electrical components with a enormous applications in electronics, especially in digital electronics. Extensive studies have been done on different types of graphene or CNTs in particular graphene nanoscroll and their applications in nanoelectronics, especially nanoscale transistors, sensors, and various devices^10,21–34^. Because of their geometry stability and engineerable chirality compare to the planar configurations, ZGNSs have been projected on new generations of nanoelectronic devices^26,35–37^. Conversely time delay as a crucial parameter in the design and implementation of CMOS technology need to be analysed. Because it is the major vital parameter in the dynamic operation of CMOS and has an inverse correlation with speed. The reduction of this parameter leads to faster switching and higher frequency in ICs^38^. In this paper by calculating and analyzing of time delay in CMOS based zigzag GNS (ZGNS) we are trying to investigate the importance of different factors to reduce time delay, resulting in faster switching and higher frequency in integrated circuits. In the following sections the proposed analytical model, the results, and the main findings of the model are presented.

## Proposed analytical model

In order to comprehend the current and time delay calculations for the same and different channel lengths of nanotransistors and identify their physical phenomena, the analytical models and results are presented in this section.

### Current calculation in nanotransistors

A general relation for current in nanotransistors states that the current directly relates to carrier concentrations (*n*_*i*_), carrier velocity (*v*), and cross-section of channel area (*A*)^39^. Carrier velocity can be calculated by Eq. (1)^40:^

1 $$v = \frac{{\mu_{n} E}}{{1 + (E/E_{crt} )}}$$

where $\mu_{n}$ is the electron (or hole) mobility and *E*_*crt*_ is the critical field which is the electrical field throughout the channel when the velocity of carriers reaches the saturation velocity. *E* is the electrical field along the channel and is considered to be half of the *E*_*crt*_. In this paper, the mobility and saturation velocity of CNT has been used instead of their amounts in GNS due to the comparable property of GNS and CNT. Therefore mobility and saturation velocity values are considered 10^5^ (cm^2^/V∙s) and 2 × 10^7^ (cm/s), respectively^41^ and the mobility as a function of E-field is unspecified. For simplicity, If we assume that the mobility doesn’t change tangible with *E*-field changes, *E*_*crt*_ can be written as Eq. (2)^41^:

2 $$E_{crt} = v_{s} /\mu_{n} = 200\;\left( {V{/}cm} \right)$$

Using the relation (1), carrier velocity (*v*) can be calculated as 7 × 10^6^ (cm/s). In numerical analysis the effects of variations for channel width are neglected and the channel length assumed to be 200 nm. In the definition of the degenerate and non-degenerate regions, the Fermi level, with a distance more than 3K_B_T from either the valence or conduction band edge within the band gap, exhibits a non-degenerate condition. Conversely, in the degenerate region, the Fermi level is within 3K_B_T of either the band edge or lies inside a band^42^. The carrier concentration of ZGNS in the degenerate region is obtained by Eq. (3)^43^:

3 $$n_{{i_{dg} }} = \frac{{4m^{*} }}{{3\pi \hbar^{2} }}\sqrt {\frac{{K_{B} T}}{\gamma }\eta_{F} }$$

where *K*_*B*_ is the Boltzmann constant, *T* is temperature, $\hbar$ is the Plank constant, γ is the overlap energy of the carbon–carbon bond which is considered 3.0 eV^44^. The normalized Fermi energy is defined as $\eta_{F} = \frac{{E_{F} - E_{C} }}{{K_{B} T}}$ and the effective mass is obtained using Eq. (4)^43^:

4 $$m^{*} = \frac{{\hbar^{2} }}{{9\gamma a_{c - c} \left[ {1 + \left( {L/2n} \right)^{2} } \right]}}$$

where *a*_*c-c*_ is the carbon–carbon bond length, *L* is the nanoscroll length, and *n* is the chirality number of nanoscroll. Moreover, the conduction energy is calculated as $E_{C} = \frac{1}{2}\left[ {\gamma + 3\left( \frac{L}{n} \right)^{2} } \right]$^43^, and *E*_*F*_ is the Fermi energy that can vary by carrier concentration but in this paper, it is considered to be constant and equal to 0.110 eV^45^ and the carbon bond length is 1.41 Å; Therefore the carrier concentration of ZGNS in the degenerate regions can can be obtained as:

5 $$n_{{i_{dg} }} = \frac{0.110}{{1 + \left( {L/2n} \right)^{2} }}\sqrt {0.5\left( {L/n} \right)^{2} - 0.463}$$

The drain current I_D_ is equal to ${n}_{{i}_{dg}} \cdot q\cdot v\cdot A$ where ${A=W.L}_{ch}$^39^. If we assume that the applied E-field is the half of the critical E-field and by using Eqs. (1) and (5), the drain current as a function of L, L_ch_ and n can be calculated as:

6 $$I_{{D_{{}} }} = \frac{{2.464 \times 10^{ - 4} }}{{1 + \left( {L/2n} \right)^{2} }} \cdot L_{ch} \cdot \sqrt {\frac{1}{2}\left( \frac{L}{n} \right)^{2} - 0.463}$$

**Figure 2:**
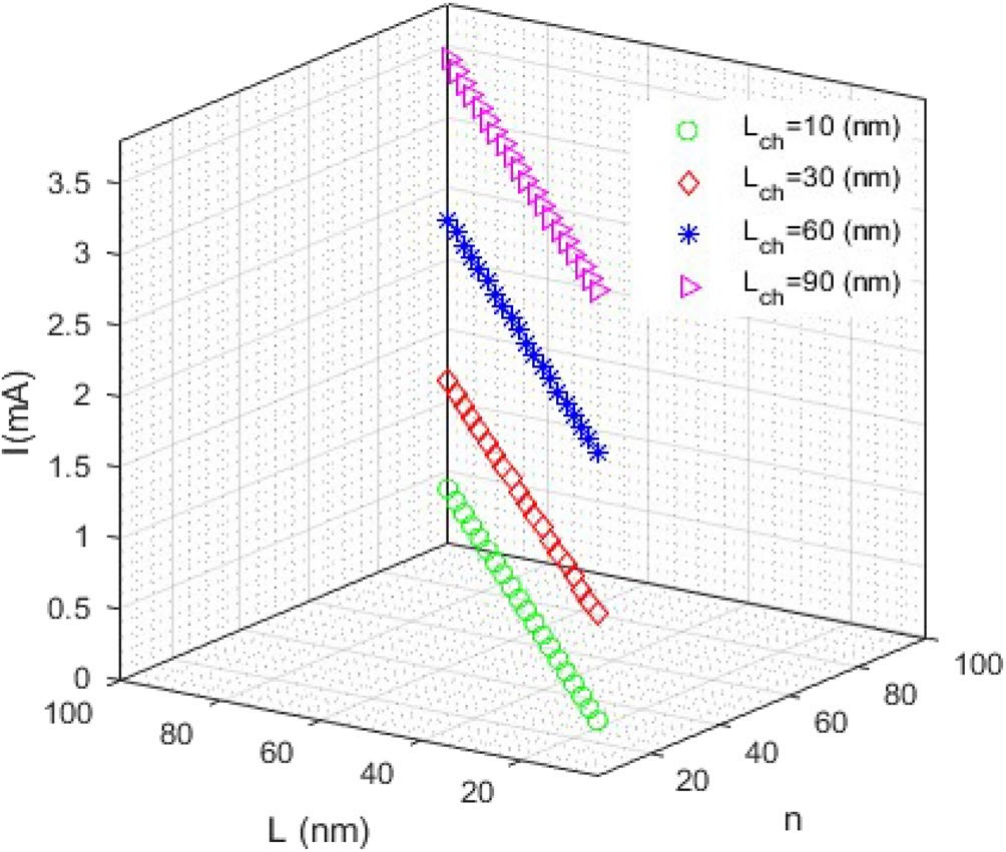
The current vs L (nm) and n: the current along the channel increases with increasing the size of the channel length.

**Figure 3:**
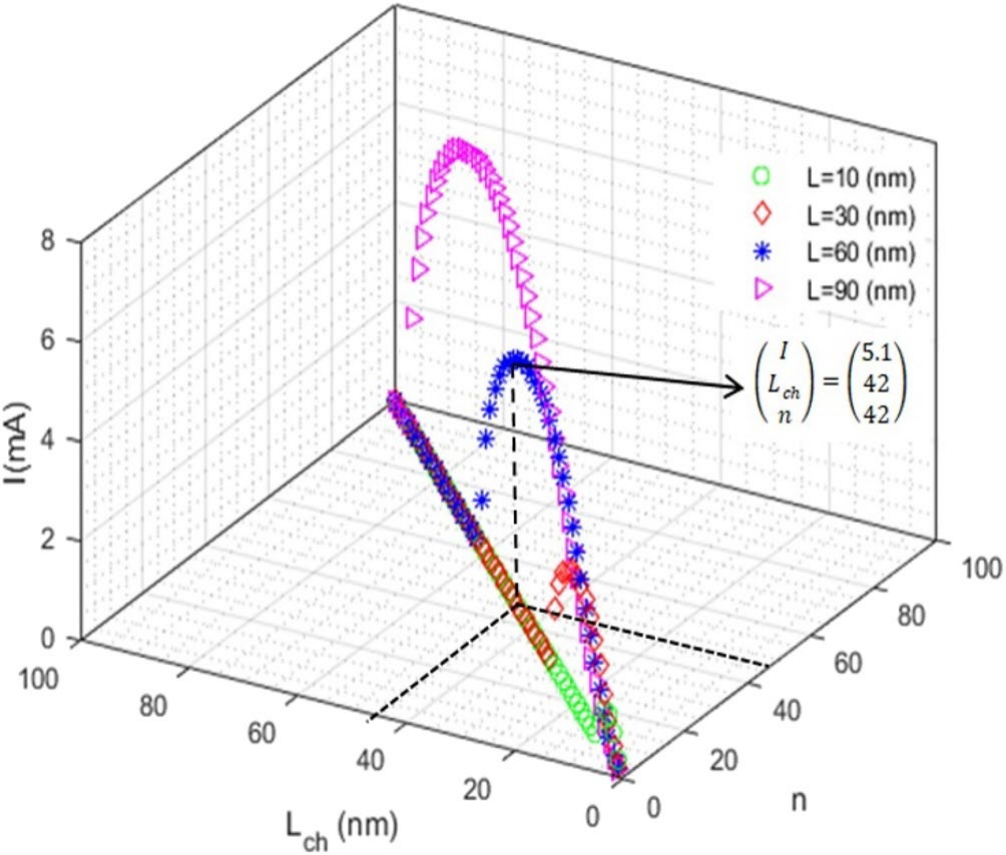
The current vs L_ch_ (nm) and n: after a special point, the curve, suddenly goes to zero. The point along L_ch_ axis is about the nanoscrolls length.

The following three-dimensional (3D) figures show the current function versus two other variables with assuming that the third variable is constant. Figure 2 shows the current curve versus *L* and *n* for several different channel lengths. As can be seen, the current along the channel increases with increasing the size of the channel length. The current behavior versus *L*_*ch*_ and *n* for different values of nanoscroll length is illustrated in Fig. 3. The curves are parabolic before a special point and after that suddenly become zero. The amount of this special point along the *L*_*ch*_ axis is approximately equal to the nanoscrolls length. For example, the current turns to become zero at *L*_*ch*_ = 42 nm for L = 60 nm.

When the carbon nanostructure is curved, tension is induced in the carbon atoms located at the edge and central part of the device therefore a barrier^46^ is shaped which directs to a p–n junction like performance in the structure. Consequently quasi-saturation behavior is expected^47,48^.

**Figure 4:**
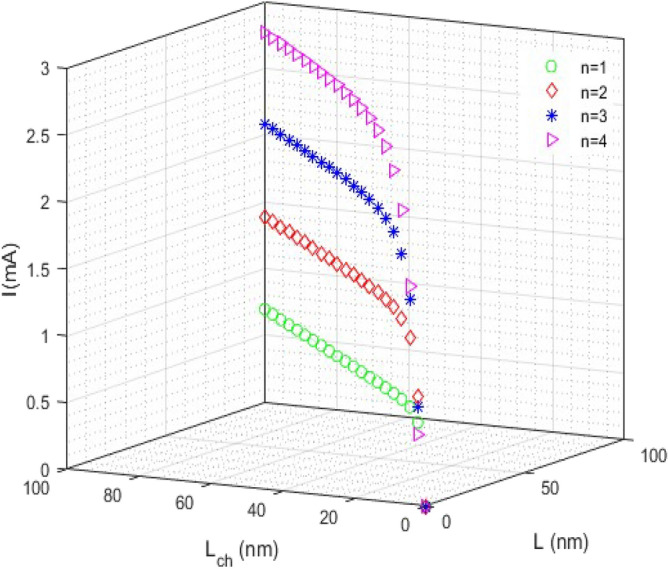
The current curve versus L (nm) and L_ch_ (nm).

Figure 4 represent the current curve versus *L* and *L*_*ch*_ for different chirality numbers. In this case, the current increases exponentially and then saturates to a special amount. Also, the current value increases with increasing the chirality number (*n*).

**Figure 5:**
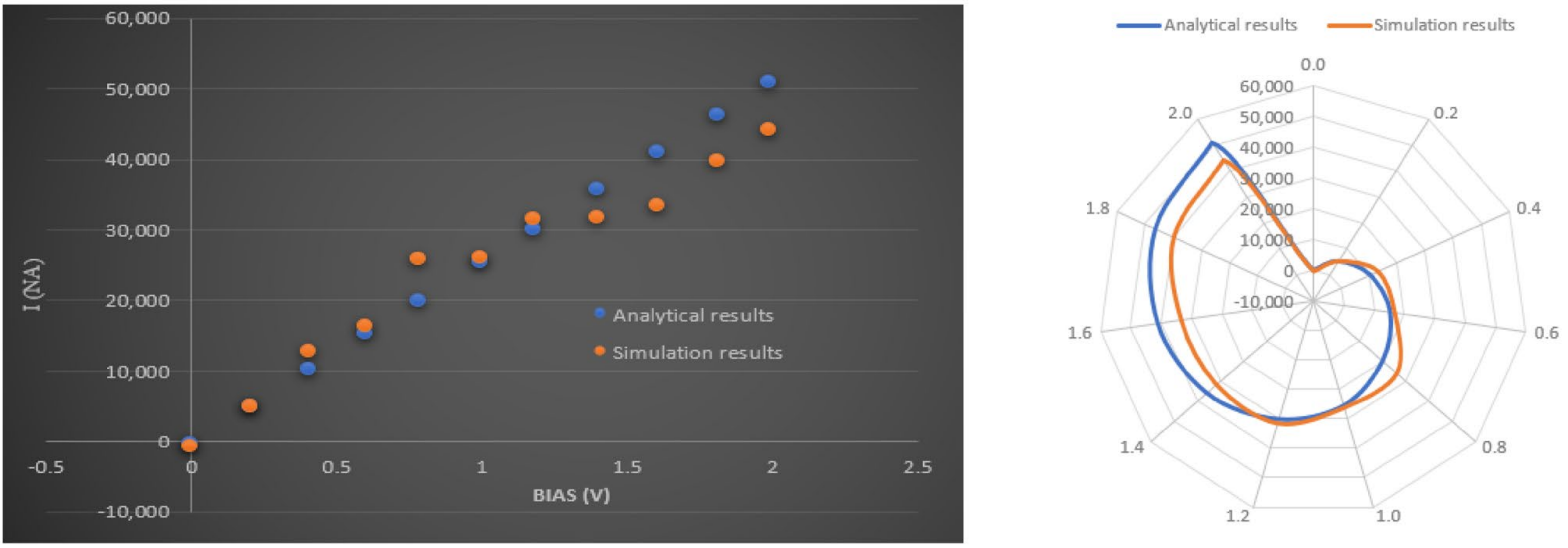
Comparison study between simulation and presented model.

In this research, only semiconductor GNSs are considered and it is notable fact to know that they are ambipolar semiconductors therefore, the relation (5) is valid in both n- and p-channel transistors for the channels with the same lengths and widths. As ZGNSs in nanoscale size dominated by quantum confinement effect in two cartesian directions, consequently, they can operate in quantum limit. So, the one dimensional current density, $J$ is equal to $\left({e}^{2}/\pi {\mathrm{\hslash }}\right){V}_{applied}{T}_{(E)}$. Also, it is expected that these devices can operate in the ballistic limit, therefore, the transmission (T) could be assumed to be 1. Additionally, considering spin selectivity in ZGNS, the current can be modified as $I=\left({e}^{2}/hA\right){V}_{applied}{T}_{(E)}$. Where, e is the electron charge, A is the cross section of ZGNS,${V}_{applied}$ is the applied voltage and h is the Plank constant. The comparison study between presented model and simulation results as shown in the Fig. 5 indicates good agreement.

### Time delay in CMOS

**Figure 6:**
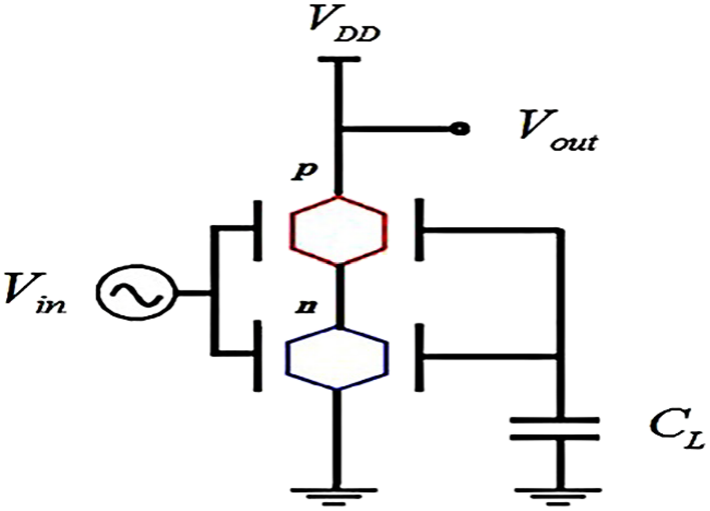
CMOS with (n and p)-channel transistors.

Time delay is an interval time between the “on” and “off” states of a transistor. This parameter is one of the important dynamic properties of CMOS inverters; Decreasing time delay causes faster transistors and consequently CMOS become faster. Figure 6 shows a CMOS that consists two n-channel and p-channel transistors which are connected in series.

*C*_*L*_ is a capacitor that is considered in small signal model analysis. *V*_*DD*_ is the applied voltage on the source terminal of the p-channel transistor. The Eqs. (7) and (8) represent the time delay for this design^49^:

7 $$\tau_{D} = \frac{{C_{L} V_{DD} }}{I}$$

where:

8 $$\tau_{D} = \frac{1}{2}\left( {\tau_{{D_{n} }} + \tau_{{D_{p} }} } \right)$$

$\tau_{{D_{n} }}$ and $\tau_{{D_{p} }}$ are the time delay of n-channel and p-channel transistors, respectively^50^. *I* is the current along the channel area of transistors. At the following section, the time delay will be studied in two cases of the same and different channel lengths.

### Time delay for same channel lengths

By using Eqs. (6), (7), and (8), the time delay for the channel lengths can be obtained as:

9 $$\tau_{D} = \frac{{C_{L} V_{DD} }}{{2.464 \times 10^{ - 4} }} \cdot \frac{{1 + \left( {L/2n} \right)^{2} }}{{\sqrt {0.5\left( {L/n} \right)^{2} - 0.463} }} \cdot \frac{1}{{L_{ch} }}$$

**Figure 7:**
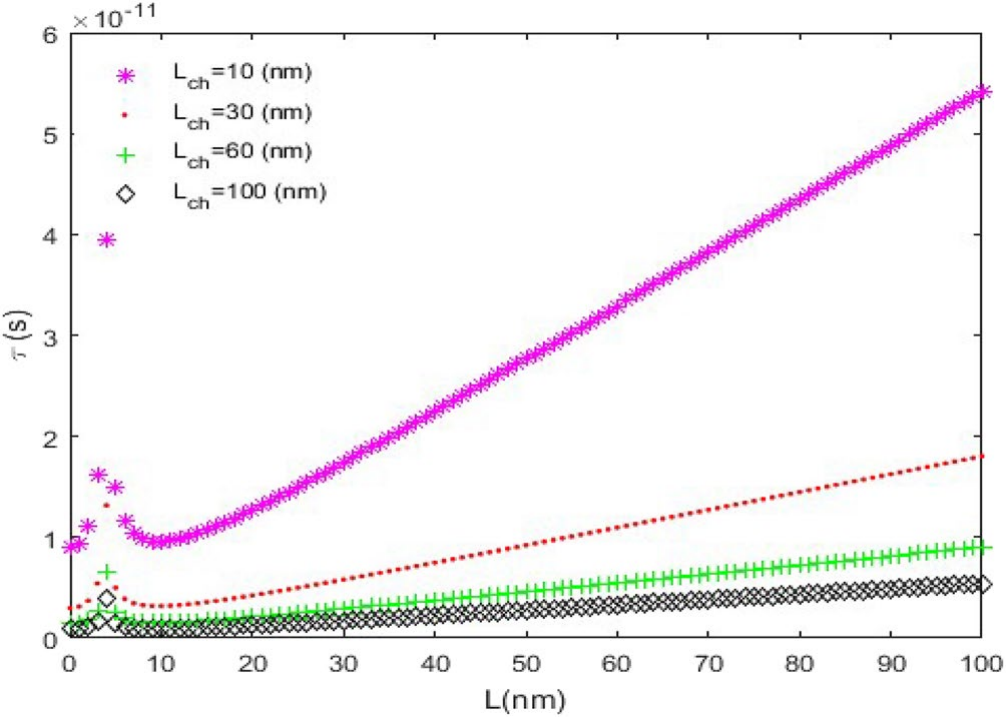
The time delay ($\tau_{D}$) versus *L*, (*n* = 4).

As can be seen in the equation, the time delay is a complex function of different parameters such as *C*_*L*_, *V*_*DD*_,* L*,* n,* and *L*_*ch*_. *C*_*L*_ and *V*_*DD*_ values are usually considered as the assumed amounts and time delay is plotted versus the other three variables. The results are plotted in 2D for the following states:

**(i) **$\tau_{D}$** versus L:** In this case, we have assumed that $C_{L} = 10fF$ and $V_{DD} = 1.5V$. Figure 7 shows the time delay of the ZGNS-based CMOS for *n* = 4 and different channel lengths. In the GNSs with equal chirality number and length, the one with the shortest channel length has the longest time delay. It is noteworthy that the diagrams for lengths of less than 10 nm lost the linear behavior. The observed trend is attributed to the quantum effects.

**Figure 8:**
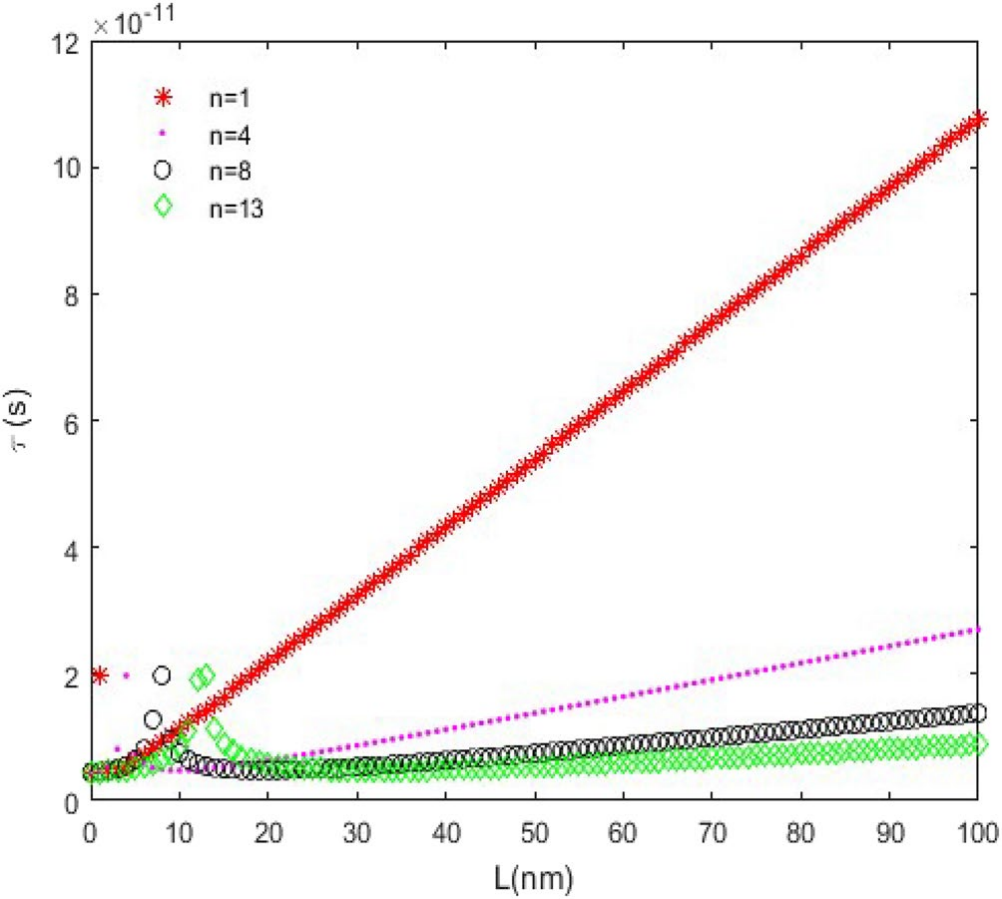
The time delay ($\tau_{D}$) curve versus *L*, (*L*_*ch*_ = 20 nm).

Figure 8 illustrates the time delay curve for *L*_*ch*_ = 20 nm and different chirality numbers. In this case, the quantum effects also appear for lengths of less than 10 nm. Among the GNSs with the same channel length, the one with the largest chirality number has the shortest time delay.

**Figure 9:**
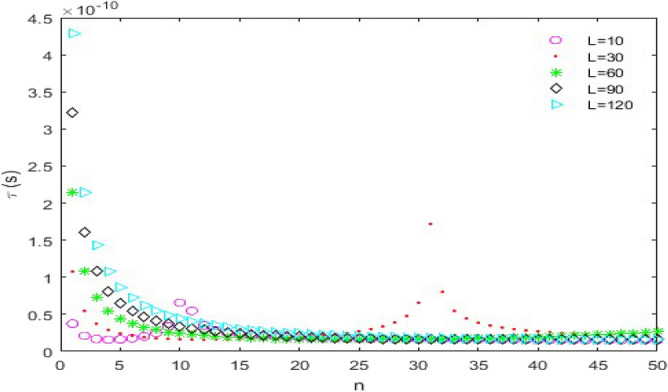
The time delay ($\tau_{D}$) versus *n*, (*L*_*ch*_ = 20 nm).

**(ii**) $\tau_{D}$ **versus n: **In this case, the following parameters have been assumed:$C_{L} = 25fF$ and $V_{DD} = 2.0V$. Figure 9 indicates the time delay of the CMOS which is plotted for *L*_*ch*_ = 20 nm and different nanoscroll lengths. The pick of time delay curves increases significantly with increasing *L*. Moreover, $\tau_{D}$ decreases exponentially with increasing *n* and that is favorable because it makes the CMOS faster. A notable point that can be understood from the diagrams is that for smaller and comparable values of *L* with channel lengths (*L* = 10 nm and 30 nm), the diagrams increase up to the pick point then decrease and do not have uniform behavior which isn’t a favorable case. For this reason, it’s suitable that use longer lengths than channel lengths to have a uniform behavior.

**Figure 10:**
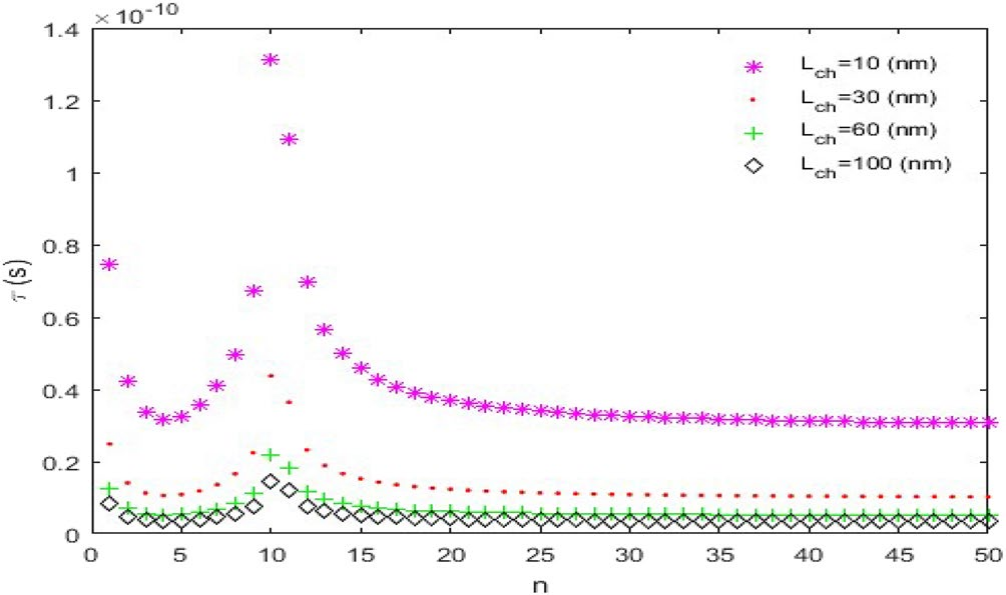
The time delay ($\tau_{D}$) versus *n*, (*L* = 10 nm).

Figure 10 is a plot of time delay as a function of *n* for *L* = 10 nm and different channel lengths. As can be seen in Fig. 10, the time delay ($\tau_{D}$) suddenly becomes zero for $n \ge 11$. In addition, among the nanoscrolls with equal lengths and chirality numbers, the one with the longest channel length has the shortest time delay.

**(iii) **$\tau_{D}$** versus L**_***ch***_**:** In this case, we have assumed that $C_{L} = 15fF$ and $V_{DD} = 1.8V$. This is a routine case; because the $\tau_{D}$ behavior versus *L*_*ch*_, is exactly similar to a reciprocal function.

**Figure 11:**
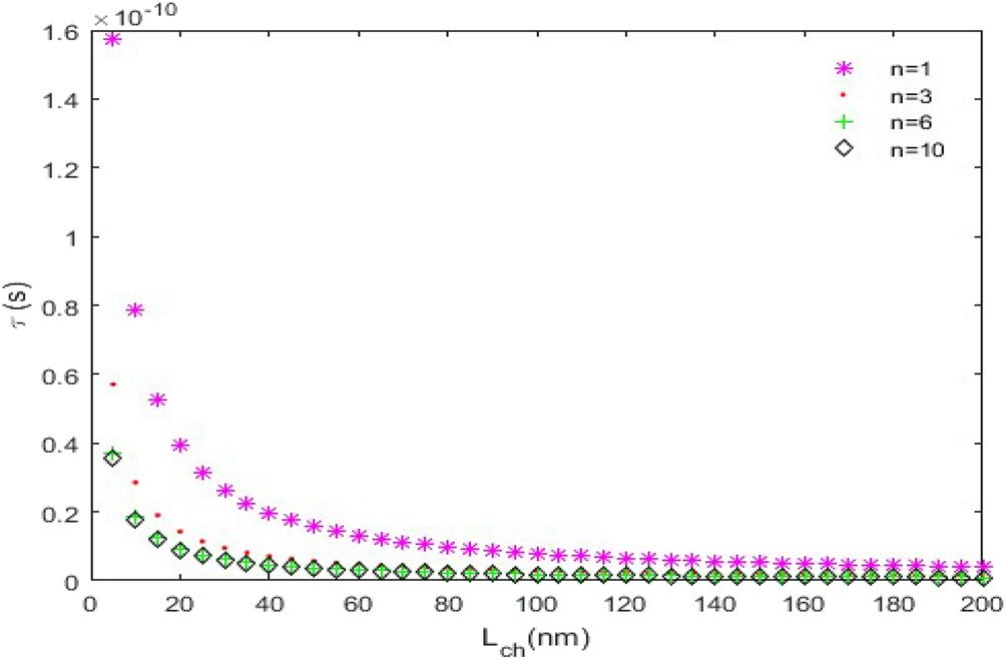
The time delay ($\tau_{D}$) versus *L*_*ch*_, (*L* = 20 nm).

Figure 11 depicts the time delay of the ZGNS-based CMOS for *L* = 20 nm and different chiralities. Among the nanoscrolls with equal lengths, the one with the biggest chirality number has the shortest time delay.

**Figure 12:**
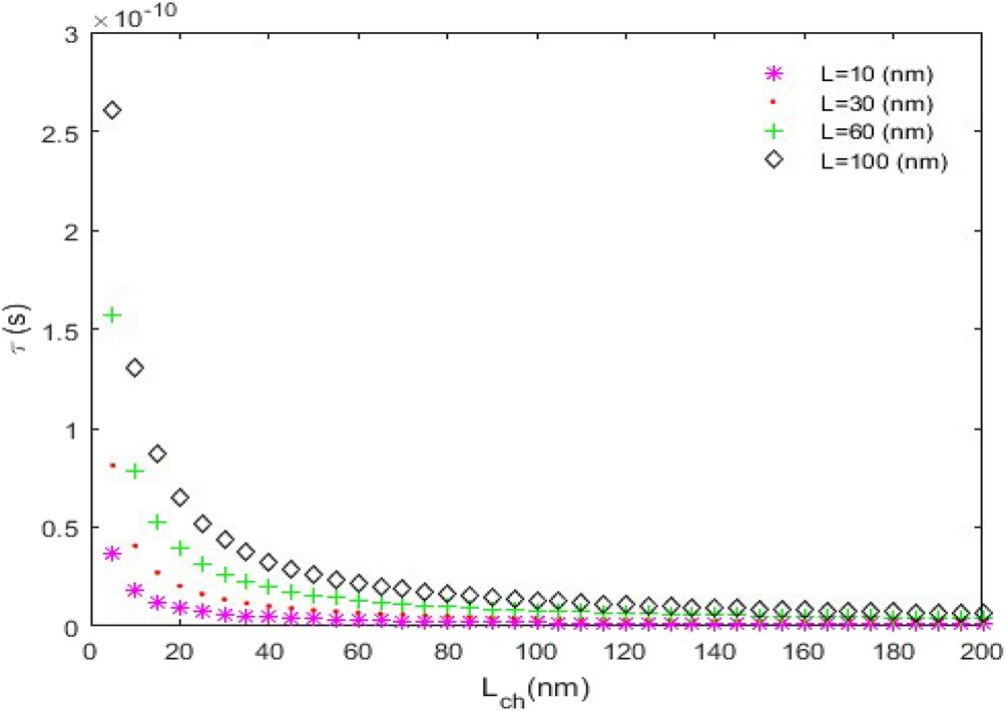
The time delay ($\tau_{D}$) versus *L*_*ch*_, (*n* = 3).

Figure 12 indicates the time delay curve for *n* = 3 and different nanoscroll lengths. Apparently, among the nanoscrolls with equal channel lengths, the one with the largest length has the longest time delay.

### Time delay for different channel lengths

In current study transistors with different channel lengths are considered, also it is assumed that multiplying of the length and the width of an n-type transistor is equal to that of a p-type transistor, i.e. ${\left({WL}_{ch}\right)}_{n}={\text{m}}{\left({WL}_{ch}\right)}_{p}$, additionally similar carrier concentration and carrier motilities for n-type and p-type transistors $\left(n=p;{v}_{p}={v}_{n}\right)$ are presupposed; furthermore, based on Eqs. (6), (7) and (8), the time delay for ${C}_{L}=15 fF$ and ${V}_{DD}=1.5 V$ can be obtained as

10 $$\tau_{D} = 9.1 \times 10^{ - 11} \frac{{1 + \left( {L/2n} \right)^{2} }}{{\sqrt {0.5\left( {L/n} \right)^{2} - 0.463} }} \cdot \frac{1}{{L_{ch} }} \cdot \left( {\frac{m + 1}{m}} \right)$$

The Eq. (10) looks like Eq. (9). The difference is just in coefficient term i.e. $$9.1 \times 10^{ - 11}$$$$\left( {\frac{m + 1}{m}} \right)$$. Therefore, the time delay for $$m > 1$$ has a little difference with a similar case compared to $$0 < m < 1$$. For example, the time delay for $$m = 0.1$$ is 11 times of a similar case in the same channels. Whereas, for $$m = 3$$, the time delay is 1.33 times of similar cases in the same channels. There are three operational possibilities for the decreasing time delay in the ZGNS-based CMOS:

1. The channel length should be increased for constant chirality number and nanoscroll length,
2. The chirality number should be increased for constant channel and nanoscroll length and
3. The nanoscroll length should be decreased for constant chirality number and channel length.

These cases are valid for both CMOS with the same and different transistors as well. Also, using CMOS with the same transistors gives a shorter time delay compared to the different transistors^51^. In this paper, the temperature effect has been neglected. Modification of some approximations may yield better and more accurate values for the time delay in ZGNS-based CMOS technology.

## Simulation study

**Figure 13:**
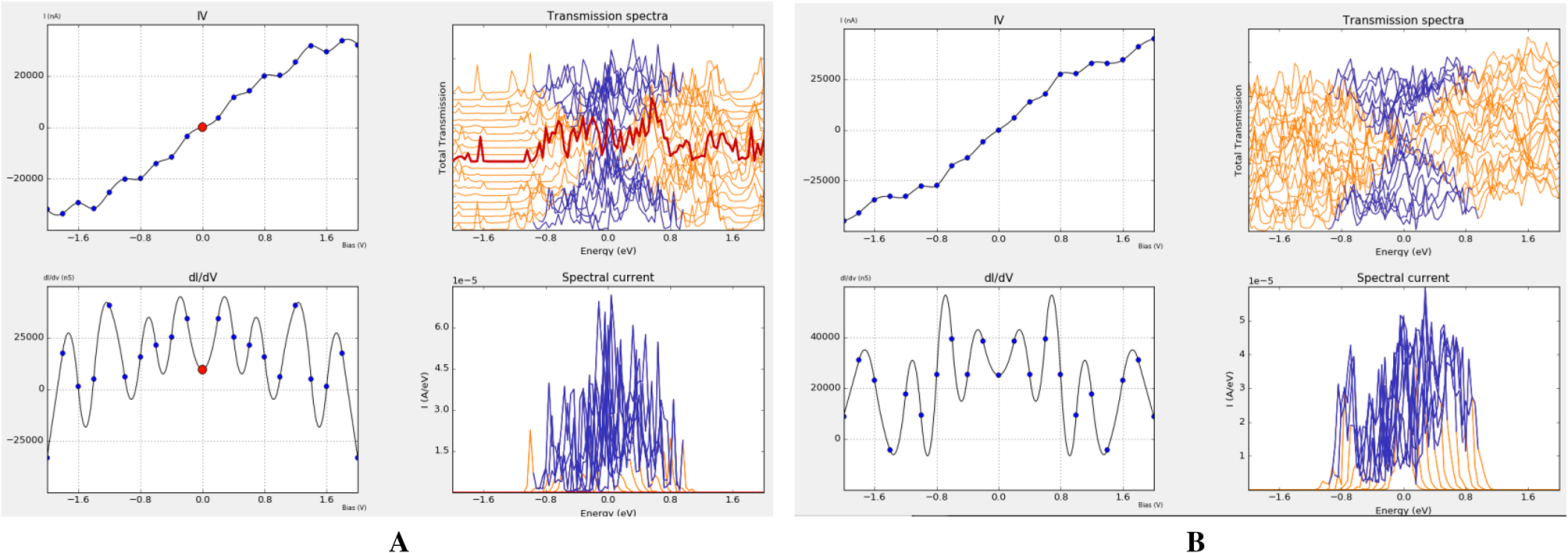
(**A**) Up-spin, (**B**) down spin characteristics.

TCAD simulation based on density functional theory (DFT) method is carried out, in the platform of the metal–semiconductor-metal structure by polarized consideration which gives us spin up and down calculation separately as shown in Fig. 13.

**Figure 14:**
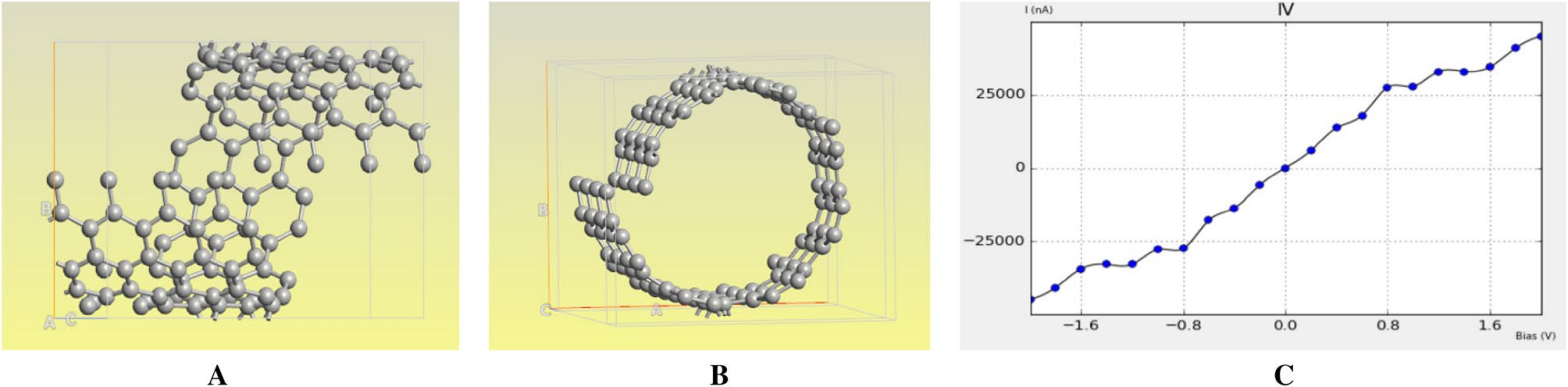
(**A**) (2, 2) graphene after 360 wrap, (**B**) with 4 Å displacement in Z direction, (**C**) Current–voltage characteristic.

To generate desired ZGNs basic (2, 2) graphene as a GNS building block with several nanometer in length is wrapped 360 degrees (Fig. 14A,B). Generated zigzag GNS is simulated based on DFT method and current–voltage characteristic is presented as shown in Fig. 14C.

**Figure 15:**
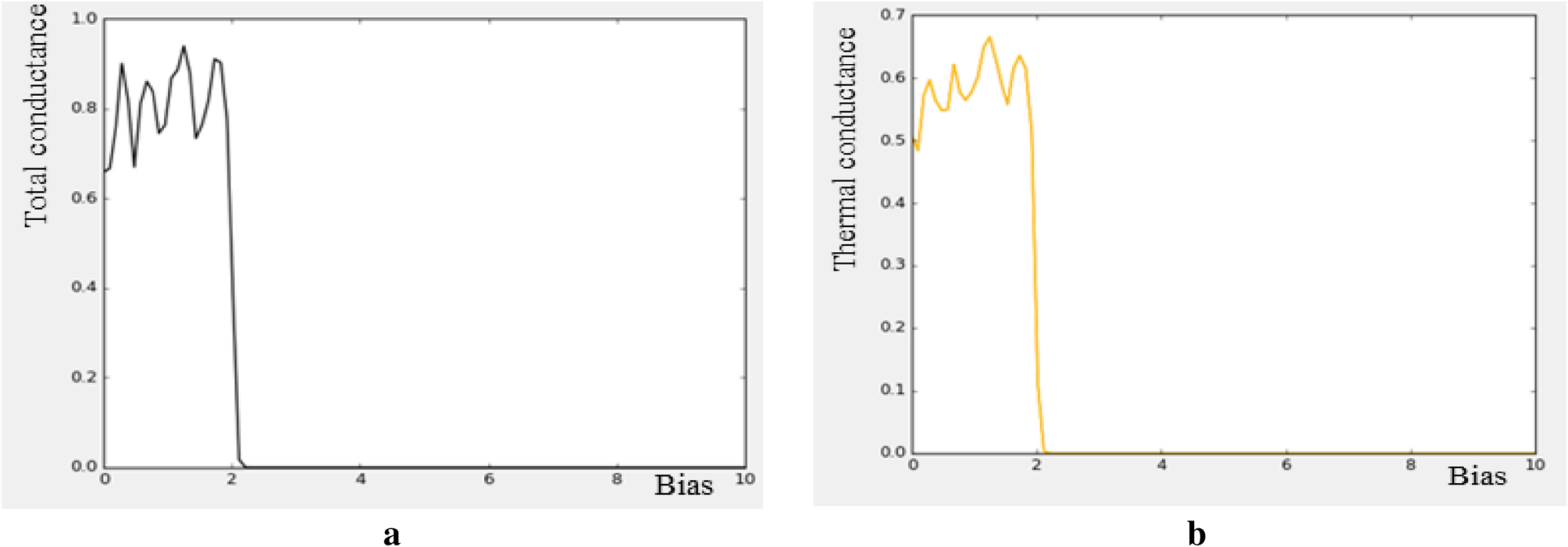
(**a**) Total conductance, (**b**) thermal conductance for ZGNS.

Additionally, TCAD simulation for total conductance and thermal conductance is explored. It needs to be notified that the total conductance is higher than the thermal conductance which indicates higher electron transport in comparison with phonon transport as shown in the Fig. 15. In the other words, less energy in the form of heat in these devices are wasted, therefore, low energy consumption is expected compared to the conventional structures.

**Figure 16:**
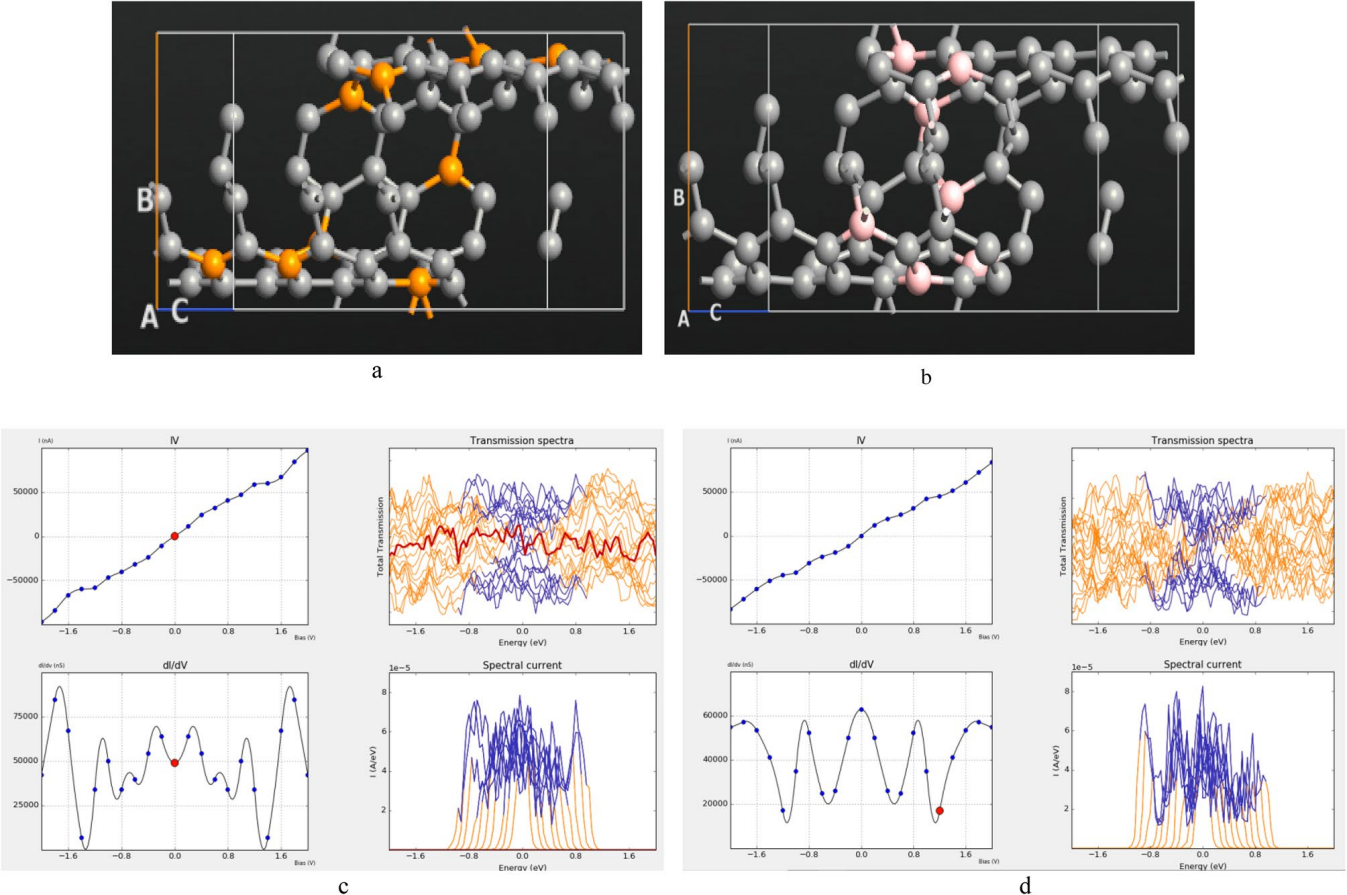
(**a**) Phosphorous doped structure, (**b**) boron doped structure, (**c**) device characteristics for P-doped structure and (**d**) device characteristics for B-doped structure.

To explore the morphology effect, Phosphorus (P) and Boron (B) in the zigzag graphene nanoscroll structure is considered and modified devices are characterized as shown in Fig. 16.

**Figure 17:**
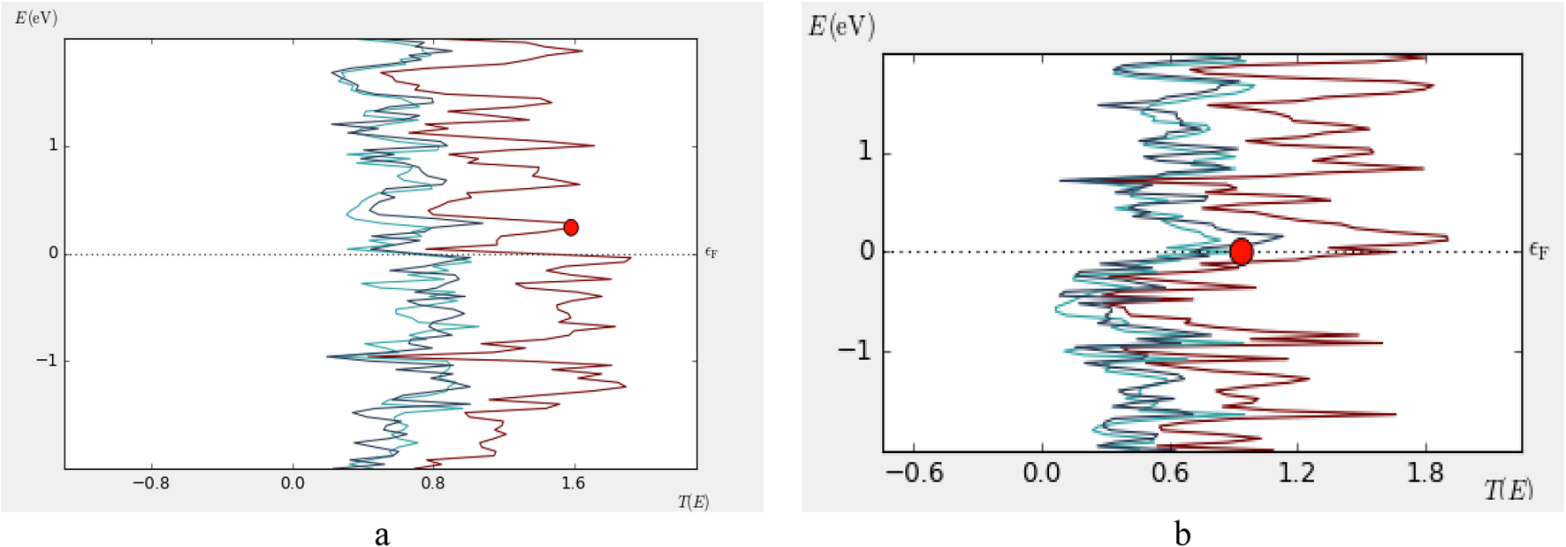
Transmission analysis in (**a**) phosphorous doped, (**b**) boron doped.

It is concluded that ZGNS indicates ambipolar behavior based on the I-V characteristic for P or B doped ZGNS structure which is in line with reported results for carbon based materials such as carbon nano tube and graphene nano ribbon. Variation of current to the bias (dI/dV) that represents the conductance in the Phosphorous doped structure in comparison with the Boron doped structure indicates dramatic disparity almost in all range of the biases. As shown in transmission spectrum (Fig. 17), by applied bias in the channel region transmission is increased in both of the cases. Transmission analysis for different ZGNS shows that Phosphorous generates available transmission states below the Fermi level but Boron generates available states right above the Fermi level.

**Figure 18:**
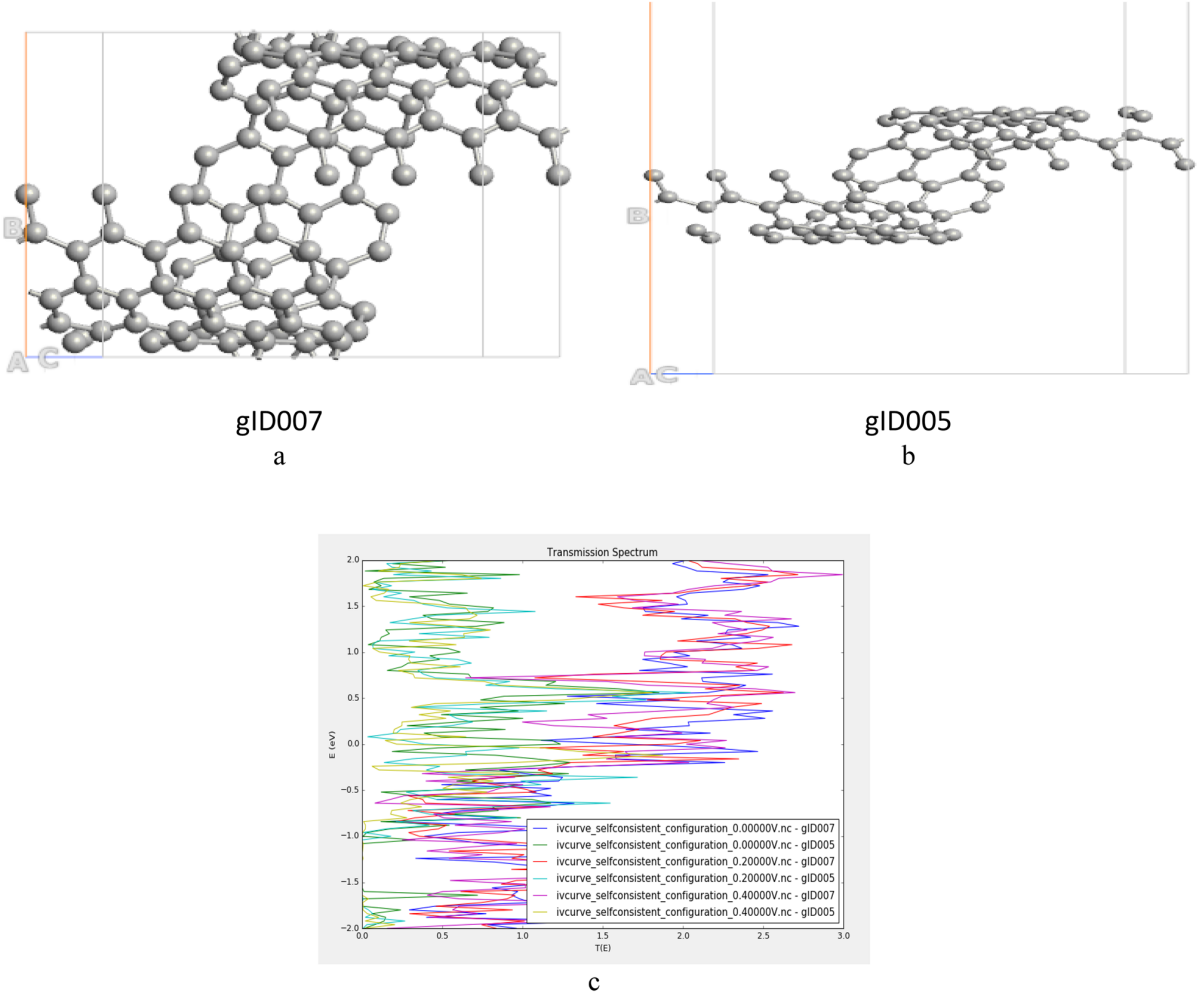
(**a**,**b**) GNS with different radii as giD007 represents bigger radius, (**c**) transmission analysis.

To investigate the size effect on GNS device performance, different radii are considered as shown in Fig. 18.

As Fig. 18c shows, for negative energy region the transmission profiles are about same but at the positive region one can clearly see that the transmission increased by radius which can be explained by existence of more rooms for electrons in bigger structure.

## Conclusion

The application of GNS in complementary metal-oxide-semiconductors (CMOS) is a promising avenue for the advancement of nanoelectronics. This study focuses on calculating and analyzing the time delay in CMOS devices that are based on ZGNS. Time delay is a crucial issue of CMOS dynamic operation and has an inverse relationship with speed. A decrease in this parameter results in faster switching and higher operating frequencies in integrated circuits (ICs). ZGNS-based CMOS shows considerable variations in the current due to parameters such as chirality number, channel length, and nanoscroll length which can be engineered to produce faster ICs. The results of this research show for n = 3 and different nanoscroll lengths, apparently, among the nanoscrolls with equal channel lengths, the one with the largest length has the longest time delay while, for n = 4 and different channel lengths, in the GNSs with equal chirality number and length, the one with the shortest channel length has the longest time delay. On the other hand for L_ch_ = 20 nm and different nanoscroll lengths, among the nanoscrolls with equal lengths and chirality numbers, the one with the longest channel length has the shortest time delay. Also for L_ch_ = 20 nm and different chirality numbers, among the GNSs with the same channel length, the one with the largest chirality number has the shortest time delay. So it can be concluded that the ZGNS-based CMOS can be engineered to increase the current via parameters such as chirality number, channel length, and nanoscroll length to get faster ICs. Finally, this study was a step ahead to shed more light on ZGNS-based CMOS devices and the chirality (zigzag, armchair and chiral), defects, stress, tension and substrate influences need to be considered in the future works.

## Data Availability

The datasets used and/or analysed during the current study available from the corresponding author on reasonable request.
